# Mechanical Support in High-Risk Pulmonary Embolism: Review Article

**DOI:** 10.3390/jcm13092468

**Published:** 2024-04-24

**Authors:** Amer N. Kadri, Razan Alrawashdeh, Mohamad K. Soufi, Adam J. Elder, Zachary Elder, Tamam Mohamad, Eric Gnall, Mahir Elder

**Affiliations:** 1 Divion of Cardiovascular Medicine, Main Line Health, Lankenau Medical Center, Wynnewood, PA 19096, USA; 2 Department of Medicine, Faculty of Medicine, University of Jordan, Amman 11942, Jordan; 3 Division of Cardiovascular Medicine, University of Texas Medical Branch, Galveston, TX 77550, USA; 4 School of Medicine, Wayne State University, Detroit, MI 48202, USA; 5 School of Medicine, American University of Caribbean, 33027 Cupecoy, Sint Maarten; 6 Heart and Vascular Institute, Detroit, MI 48201, USA; 7 College of Osteopathic Medicine, Michigan State University, East Lansing, MI 48864, USA; 8 Corewell Health East, Dearborn Hospital, Dearborn, MI 48124, USA

**Keywords:** pulmonary embolism, right ventricular failure, mechanical support, hypoxia

## Abstract

Acute pulmonary embolism (PE) may manifest with mild nonspecific symptoms or progress to a more severe hemodynamic collapse and sudden cardiac arrest. A substantial thrombotic burden can precipitate sudden right ventricular strain and failure. Traditionally, systemic thrombolytics have been employed in such scenarios; however, patients often present with contraindications, or these interventions may prove ineffective. Outcomes for this medically complex patient population are unfavorable, necessitating a compelling argument for advanced therapeutic modalities or alternative approaches. Moreover, patients frequently experience complications beyond hemodynamic instability, such as profound hypoxia and multiorgan failure, necessitating assertive early interventions to avert catastrophic consequences. The existing data on the utilization of mechanical circulatory support (MCS) devices are not exhaustive. Various options for percutaneous MCS devices exist, each possessing distinct advantages and disadvantages. There is an imminent imperative to develop a tailored approach for this high-risk patient cohort to enhance their overall outcomes.

## 1. Introduction

Pulmonary embolism (PE) ranks as the third highest contributor to cardiovascular fatalities in the United States, following acute myocardial infarction and stroke. It is estimated to occur in 390,000 cases annually [1]. The presentation of PE is heterogeneous, encompassing asymptomatic cases to instances of sudden death. The contemporary risk stratification models recommended by both the European Society of Cardiology and the American Heart Association rely on the use of biomarkers, identifying systemic hypotension, and right ventricular dysfunction. In these models, individuals at high risk (also called “massive”) are characterized by hypotension and end-organ hypoperfusion (altered mental status, cold, decreased urine output, lactic acidosis), the need for vasopressors, or experiencing cardiac arrest [2,3,4]. The incidence rate of high-risk PE varies depending on the evaluated cohort, with multicenter studies estimating rates between 3% to 7%, and larger single institutional series reporting rates of up to 12% [4,5,6,7,8]. The prognosis of PE is most associated with the extent of symptomatic hemodynamic instability and asymptomatic right ventricle (RV) dysfunction. Cardiogenic shock occurs in fewer than 5% of PE patients, and the mortality rates for those with cardiogenic shock range from 25 to 40%. Furthermore, in patients needing cardiopulmonary resuscitation, the mortality rate can be as high as 65–95% [5,9].

## 2. Mechanism of Hemodynamic Collapse

Due to its thin-walled and compliant nature, the RV is less equipped to handle sudden increases in afterload [10]. In PE, the RV afterload increases acutely, with emboli obstructing 50–75% of the pulmonary vasculature, particularly in patients with pre-existing cardiopulmonary disease [11]. The afterload is further worsened with pulmonary vasoconstriction secondary to hypoxia, induced by the emboli [12]. Once afterload reaches a critical level, the RV undergoes dilation, which shifts the interventricular septum leftward, causing the left ventricle (LV) to be inadequately filled and resulting in reduced supply to the coronary arteries. This leads to a decrease in perfusion to the right ventricle as a consequence of both reduced output to the coronary arteries and heightened intramuscular pressure hindering coronary artery flow, ultimately resulting in right ventricular ischemia [13,14].

Furthermore, the elevated RV end-diastolic pressure seen in PE may increase coronary venous pressure, ventricular wall stress, and oxygen demand [14]. With the onset of ischemia, the RV contractility diminishes, leading to a further decline in the RV output. This exacerbates right ventricular dilatation and contributes to a reduction in LV output, initiating a hemodynamic spiral that intensifies and culminates in cardiogenic shock [15]. Furthermore, the induction of general anesthesia and the intubation of patients with massive PE results in hemodynamic collapse in up to 19% [16]. Importantly, normotensive patients with RV dysfunction make up 30–40% of patients presenting with PE. Among this group, 10% go on to develop PE-related shock after admission [17]. This highlights the presence of significant latent hemodynamic impairment among patients with PE. This means that a patient may seem clinically stable initially, but subsequent hemodynamic compromise can swiftly develop as the RV starts to fail under the elevated afterload conditions [12].

## 3. Treatment Options for High-Risk PE

Published guidelines outline the treatment strategies for acute PE, encompassing recommendations for patients with high-risk PE and cardiogenic shock. However, the evidence base for the management of these patients is limited, and there is a lack of consensus regarding the appropriate approach for treating acute RV-failure-induced cardiogenic shock in the setting of PE [4,5]. Anticoagulation stands as the primary treatment for PE. Nevertheless, the occurrence of adverse outcomes in patients with high-risk and intermediate-risk PE has led to the adoption of therapeutic escalation. The definitive treatment of PE aims to preserve perfusion to the lungs; examples of such treatment are anticoagulation and reperfusion therapy, which includes systemic thrombolytics, catheter-based therapy and even extracorporeal membrane oxygenation (ECMO) [9]. A detailed discussion of these therapies is beyond the scope of this review article. In cases where there is evidence of hemodynamic compromise, the existing guidelines for managing acute RV failure advocate for fluid resuscitation as the initial step [18]. However, excessive fluid administration may lead to significant RV dilatation, a rapid decline in RV performance, and the bowing of the ventricular septum, thereby compromising LV filling [19,20]. Second-line management involves providing inotropic or vasopressor support, with no specific agent recommended in the consensus guidelines [2,19,20].

## 4. Mechanical Circulatory Support (MCS)

The utilization of MCS as a rescue therapy in patients with sustained obstructive shock and as prophylactic in patients who are hemodynamically stable but at risk of shock (clues includes RV function and troponin) is crucial [19,21]. The timely implementation of MCS is essential for its success, as it plays a pivotal role in optimizing patient outcomes and preventing avoidable morbidity and mortality [18]. Once the decision is made for advanced RV mechanical support, multiple options are available. Furthermore, one must consider if the patient has a very poor pre-existing functional status or poor life expectancy that precludes the patient from receiving escalated RV mechanical support [2].

**Figure 1:**
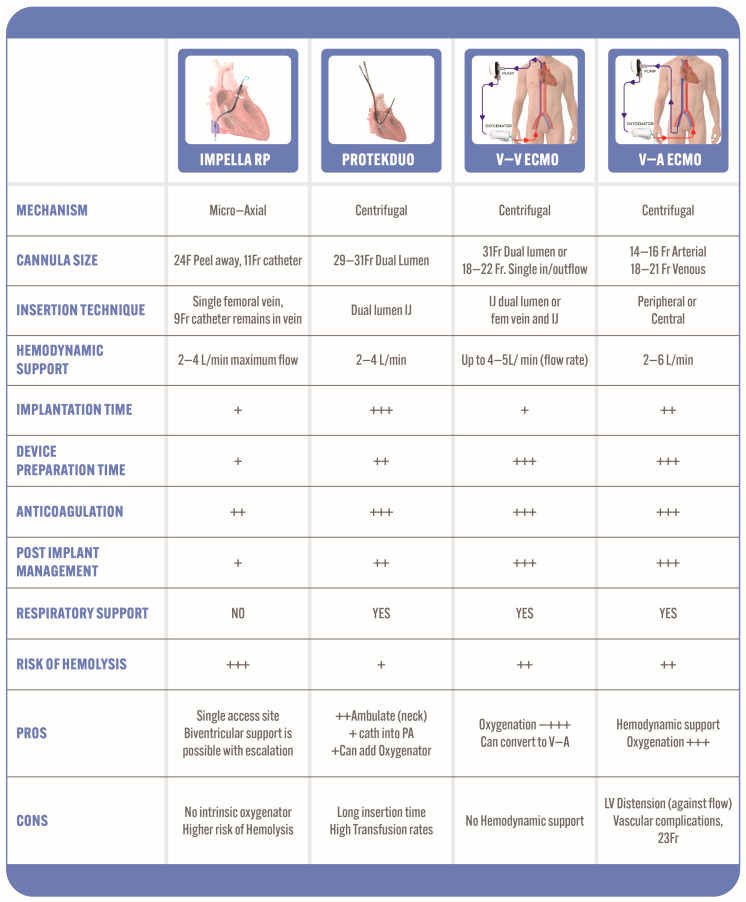
Characteristics of commercially available mechanical circulatory support devices. CVF: common femoral vein; ECMO: extracorporeal membrane oxygenation; IJ: internal jugular; LV: left ventricle; V-A: veno-arterial; V-V: veno-venous.

Various options for percutaneous MCS devices exist, each possessing distinct advantages and disadvantages (Figure 1).

## 5. ECMO

An extracorporeal circuit employed in ECMO utilizes a semipermeable membrane for gas exchange. Deoxygenated blood is drawn through a venous drainage cannula by an external continuous flow centrifugal pump, traverses the oxygenator, and is then reintroduced to the patient through an arterial (or less frequently venous) cannula. ECMO offers advantages like the ability to be percutaneously applied at the bedside for immediate emergency support. Additionally, it provides biventricular support and addresses the pathogenesis of the pulmonary system, which may not be tackled with isolated RV support [2,12,19].

**Figure 2:**
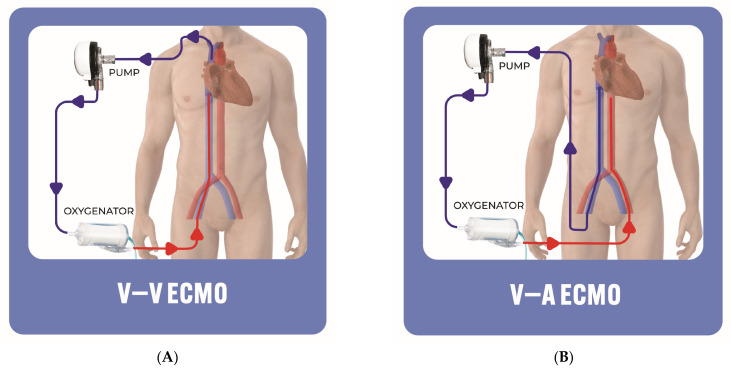
Extracorporeal membrane oxygenation: (**A**) V-V, (**B**) V-A. ECMO: extracorporeal membrane oxygenation; V-A: veno-arterial; V-V: veno-venous.

Operators have the flexibility to select the size of the cannula, influencing the level of flow within the system. In the case of veno-venous (V-V) ECMO (Figure 2A), where blood is drained from a large central vein through an outflow cannula into a peripheral oxygenator and returned to another central vein through an inflow cannula, the device is primarily geared toward providing gas exchange. V-V ECMO only provides pulmonary support in patients with severe hypoxemia refractory to invasive mechanical ventilation, and does not provide direct hemodynamic support. Furthermore, the implementation of V-V ECMO improves both the oxygenation and CO_2_ clearance, which can potentially lower the pulmonary artery vascular resistance, relieving RV strain and subsequently improving the RV function [22,23]. Therefore, it is not routinely used in catastrophic PE presenting with cardiogenic shock or cardiac arrest. Conversely, veno-arterial (V-A) ECMO (Figure 2B) involves draining blood from the venous system and pumping it into an artery bypassing the LV, delivering both respiratory and circulatory support at more than 5 L per minute [24,25]. V-A ECMO efficiently circumvents the pulmonary circulation, leading to a reduction in the RV preload and subsequently enhancing RV function [26]. ECMO, by stabilizing the hemodynamic parameters and enhancing oxygenation, can act as a bridge to other therapeutic interventions, including catheter-directed therapies and recovery [27]. Most patients presenting with PE alongside severe refractory hypoxemia have significant hemodynamic compromise, and accordingly, these patients will require V-A ECMO, not V-V ECMO. Nevertheless, V-A ECMO may elevate the LV afterload, LV wall stress and oxygen demand [26,28].

Although ECMO has been used as a cardiopulmonary hemodynamic support for stabilizing high-risk patients routinely in recent decades, there are no randomized controlled trials comparing mechanical circulatory support to the standard of care. The effectiveness of ECMO in improving survival rates among patients with high-risk PE remains uncertain. Furthermore, it is unclear which specific patients would derive the greatest benefit from this highly invasive therapy, and for whom this treatment option, with its associated major complication rate, might not be suitable. In a recent retrospective study, it was found that V-A ECMO alone or as part of a multi-pronged reperfusion approach including embolectomy or thrombolysis might offer survival benefits compared to thrombolysis alone in patients with high-risk PE [29]. In a recent extensive meta-analysis comprising 39 observational studies involving 6409 patients receiving ECMO for high-risk PE, it was reported that approximately 40% of patients receiving ECMO for PE died, mostly among those who suffered from cardiac arrest either prior to or during ECMO [30]. Patients managed with ECMO and catheter-directed therapy had significantly lower mortality (28.6%) when compared to those managed with ECMO in conjunction with systemic thrombolysis (56.99%). Moreover, patients who were treated with concurrent catheter-directed therapies had comparable outcomes to those treated with ECMO as a standalone strategy in the same study [30].

The European Society of Cardiology suggests the consideration of ECMO In conjunction with surgical embolectomy or catheter-directed treatment for refractory circulatory collapse or cardiac arrest (Level iIb evidence) [31]. In 2023, the American Heart Association issued a scientific statement examining the utilization of ECMO in high-risk PE. The statement acknowledged the challenge of assessing the certainty of evidence regarding ECMO as a mechanical circulatory support device in high-risk PE unless it is employed early, rather than as a salvage therapy [32].

ECMO cannulas are preferably inserted in awake, non-intubated patients to avoid the further worsening of hemodynamic instability related to anesthesia and mechanical ventilation. In patients with high-risk PE, the swiftest stabilization is frequently achieved by percutaneously inserting V-A ECMO with a 21–28 F venous drainage cannula into the right common femoral vein (or right internal jugular vein), coupled with an arterial cannula (15–19 F) in the left common femoral artery [33,34]. It must be noted that manufacturers have different cannula sizes. In cases where additional right-sided decompression is necessary, a second venous inflow cannula can be non-emergently placed in the right internal jugular vein. This is particularly important in patients with severely impaired upper body oxygenation following peripheral V-A ECMO cannulation [29]. To mitigate lower-extremity ischemia related to the large inflow arterial cannula, a 7 F antegrade reperfusion cannula is placed in the superficial femoral artery. The ECMO flow is adjusted incrementally until the right ventricle is decompressed, as observed through transthoracic echocardiography. ECMO blood flow is usually maintained at 2–3 L/min. Nevertheless, a certain level of pulmonary blood flow should be sustained to maintain pulmonary artery pulsatility to enable the fibrinolysis of the thrombus [35].

**Figure 3:**
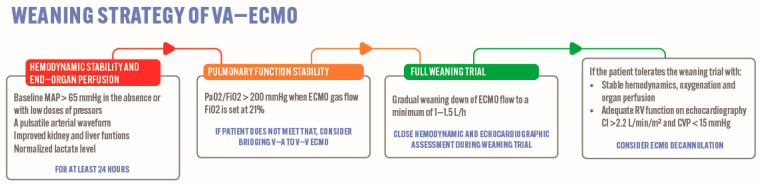
Weaning strategy of V-A ECMO. CI: cardiac index; CVP: central venous pressure; ECMO: extracorporeal membrane oxygenation; FiO_2_: fraction of inspired oxygen; MAP: mean arterial pressure; PaO_2_: partial pressure of oxygen; RV: right ventricle; V-A: veno-venous.

Supportive care with ECMO should be continued until the optimization of end-organ function and after 3 to 5 days of heparin therapy to allow for potential endogenous fibrinolysis [36,37]. The patients should be hemodynamically stable (i.e., they should maintain a mean arterial pressure (MAP) of >60 mmHg in the absence or at low doses of vasopressors or ionotropic agents, along with a pulsatile arterial waveform) for at least 24 h, have adequate perfusion parameters (such as normalized lactic acid level, liver and kidney function tests), and have pulmonary oxygenation (i.e., the ratio of the partial pressure of oxygen to the fraction of inspired oxygen should be more than 200) before the ECMO weaning trial is considered (Figure 3) [38]. In patients deemed ready for weaning, the pump flow should be weaned down by 0.5–1 L/min until ≤1.5 L/min. This raises the preload, allowing an evaluation of the cardiac recovery. The RV function evaluation should be performed with the ECMO flow turned down to evaluate the function with the RV loaded [38,39]. The suggested hemodynamic target values prior to ECMO decannulation, based on the currently available data, are as follows: right atrial (RA) pressure ≤ 15 mmHg, pulmonary arterial (PA) mean pressure and PA/RA pressure ≥ 1.5, MAP ≥ 65 mmHg, and pulse pressure ≥ 30 mmHg [39,40].

- Pros: VA-ECMO provides robust cardio-pulmonary support. Furthermore, it does not need fluoroscopic guidance for insertion, hence it can be initiated relatively quickly, offering prompt stabilization in rapidly deteriorating patients and those presenting with cardiac arrest [41,42].
- Cons: ECMO is associated with major complications that strongly influence the recovery and prognosis of patients, including vascular access complications, hemolysis, bleeding, and stroke, which are secondary to cardioembolic or cerebral hypoperfusion [43]. In addition, there is a risk of worsening LV function or LV distention, particularly in patients with pre-existing LV systolic dysfunction. Hence, various LV venting techniques can be implemented to decompress the LV in high-risk patients [44,45].

## 6. Right Ventricular Assist Device (RVAD): Right-Sided Impella Device, Impella RP (Abiomed Inc., Danvers, MA, USA)

In a multicenter study including 30 patients with RV failure (due to an AMI, after cardiotomy, or after left ventricular assist device implantation), the cardiac index significantly improved after the device placement, with an overall 30-day survival of 73% [46]. Despite a lack of enough supporting evidence, the preliminary data derived from case reports seems to be promising [28,47,48,49,50]. Hence, attempting systemic thrombolytic and mechanical thrombectomy in patients with refractory RV failure and multiorgan failure can be considered heroic measures and, thus V-A ECMO would not be indicated.

**Figure 4:**
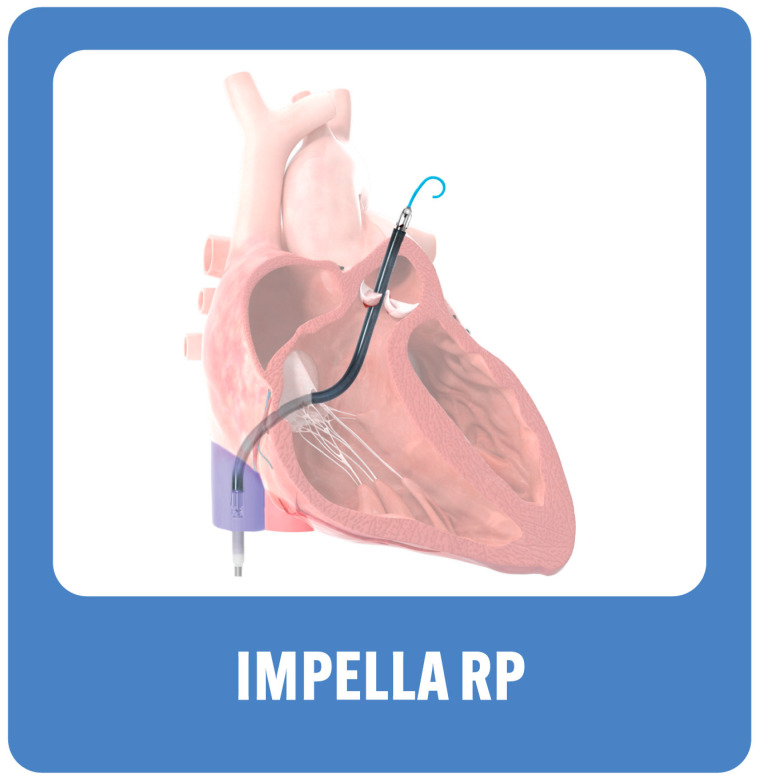
Impella RP.

The device is usually inserted into the right femoral vein over a stiff guide wire to the inferior vena cava and across the right atrium, right ventricle and to the pulmonary artery (Figure 4). The catheter itself is a 11 French in size (through a 23 F peel away sheath). It delivers blood flow up to 4 L/min. The device does not provide oxygenation as it aspirates blood from the RA to the PA, bypassing the RV. Heparin has to be given with a target activated clotting time of 160–180 s; an alternative is to use a direct thrombin inhibitor.

- Pros: The Impella RP offers easy insertion through a single venous access point, without the need for perfusionist support. The risk of hemolysis is mild. Additionally, a left-sided Impella can be added for LV support.
- Cons: The Impella RP does not provide pulmonary support with oxygenation. It cannot be used in patients with abnormal pulmonary artery anatomy as it precludes the placement of the device, those with right-sided mechanical valves or severe stenosis or regurgitation, anatomical limitations of the inferior vena cava that preclude the advancement of the device to the right atrium, or thrombus in the inferior vena cava or the right atrium.

Furthermore, it has to be inserted in the cath lab under fluoroscopic guidance. Finally, the intracorporeal motor poses a higher risk of pump thrombosis and hemolysis, which may necessitate transfusion.

## 7. Right Ventricular Assist Device (RVAD): ProtekDuo (Livanova, UK)

**Figure 5:**
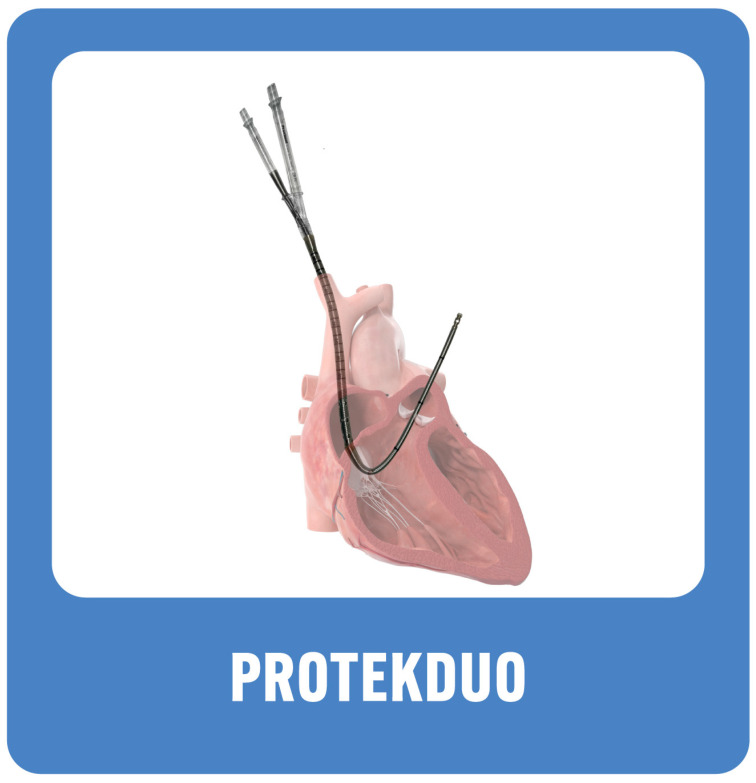
ProtekDuo.

The utilization of the ProtekDuo has been found to be safe and feasible for supporting the RV post LVAD insertion [51]. While data on its utilization in acute PE complicated by RV failure due to obstructive shock are limited, it shows promise in unloading the RV post catheter-based therapy for clot burden reduction in cases of sustained RV failure (Figure 5) [52,53].

The ProtekDuo is a single venous access temporary percutaneous RVAD that is composed of a dual lumen cannula that comes in two sizes: 29 Fr, with a distal lumen of 16 Fr and 46 cm, and 31 Fr, with a distal lumen of 18.5 Fr and 26 cm or 51 cm. The device is inserted over a stiff guidewire through the internal jugular vein, across the RA, RV and then to the main PA. The inflow cannula is positioned in the RA, transmitting blood via a centrifugal pump to the outlet cannula in the PA. If necessary, the pump can be connected to an oxygenator as well. Like other mechanical support devices, contraindications include anatomical variation that precludes the insertion of the cannula and thrombosis of the internal jugular vein or the RA. The careful selection of the cannula size is warranted.

Weaning: incremental reduction in the flow by 0.5 L/min down to 2 L/min, followed by careful assessment of the hemodynamics (such as PA pulsatility index and cardiac index), RV function by echo (such as tricuspid annular plane systolic excursion), and appropriate perfusion parameters [49]. Once the patient is deemed stable, the cannula can be removed and, if needed, a low-dose pressor can be used.

- Pros: single access via internal jugular vein, patient can ambulate if needed, and an oxygenator can be added if necessary. Less risk of hemolysis.
- Cons: Insertion requires careful measurement and time investment. Similar to Impella RP, it needs fluoroscopic guidance; thus, insertion has to be performed in the cath lab.

## 8. Special Considerations and Future Directions: Proposed Management Approach

**Figure 6:**
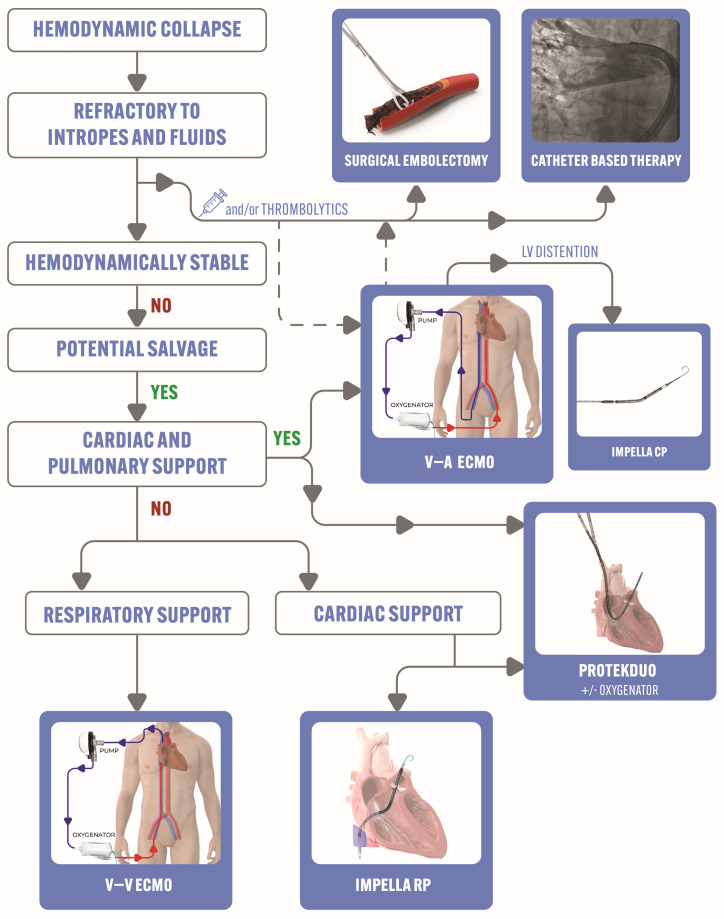
Proposed approach for managing high-risk pulmonary embolism with hemodynamic collapse. ECMO: extracorporeal membrane oxygenation; IJ: internal jugular; LV: left ventricle; V-A: veno-arterial; V-V: veno-venous.

Due to a glaring lack of high-quality data, the latest European Society of Cardiology guidelines and American Heart Association consensus statement conspicuously downplay the pivotal role of catheter-based therapy and MCS [2,31,32]. When the patient inevitably deteriorates after systemic thrombolytics (or when contraindicated) and demands more than one pressor, it is crucial to actively seek an alternative approach. We assert a streamlined algorithm, poised to markedly enhance outcomes in this acutely distressed yet potentially salvageable group. This is shown in Figure 6.

In managing patients with acute PE and hemodynamic instability, initial interventions involve intravenous fluids and low-dose pressors, with consideration given to thrombolytic therapy if no contraindications are present. If hemodynamics remains compromised despite these measures, embolectomy (either surgically or via percutaneous catheter-based therapy) may be pursued. Transthoracic echocardiogram is a crucial tool as it may detect right ventricular pressure overload and dysfunction caused by acute pulmonary emboli, and help in the risk stratification of PE [54]. In addition, echocardiogram holds a significant value in patients with suspected PE and high pretest probability, particularly when they are hemodynamically unstable and unable to undergo CT angiography, given its ability to detect certain findings of RV dysfunction that may warrant emergency reperfusion or catheter-directed therapies [31]. Although echocardiogram may provide an adequate assessment of the pulmonary vasculature hemodynamic, as well as the cardiac output and cardiac index, it can be limited by technical challenges and certain clinical situations that may render hemodynamic measurements suboptimal or inaccurate [54]. On the other hand, the upfront use of invasive hemodynamic and PA catheters in high-risk PE may offer several potential theoretical advantages; these include (1) the timely diagnosis of PE-related cardiogenic shock through the accurate measurement of filling pressures, pulmonary vascular pressures and cardiac output, and (2) the prompt initiation of hemodynamically guided MCS with serial follow-up evaluations to guide decisions regarding device escalation or de-escalation [55,56]. Nonetheless, there are no randomized controlled trials that have prospectively studied the utility of PA catheter use among patients with acute PE–cardiogenic shock.

In cases where both pulmonary and cardiac support are necessary, V-A ECMO is the preferred approach (which can also serve as a bridge to surgical embolectomy if required). In the event of left ventricular failure or distention, consideration may be given to utilizing a left-sided MCS device such as Impella CP. For stable patients with pulmonary insufficiency characterized by hypoxia or hypercapnia, V-V ECMO can be considered. In situations where there is a low residual burden of pulmonary embolism and sole cardiac support is needed, options like Impella RP^®^ or ProtekDuo (which can be seamlessly connected to an oxygenator for additional pulmonary support) should be considered.

This approach serves as a guiding force for the dedicated PE response team during their discussions. Ideally, this team should encompass the leading interventional cardiologist or radiologist, critical care and heart failure/shock specialists, as well as the cardiothoracic surgeon. The formation of such a well-coordinated team, where possible, enhances the efficiency and effectiveness of the treating facility, especially when considering the transfer of high-risk patients from nearby facilities. This transformation raises the center’s status to a high-volume facility, known for delivering excellent outcomes [57,58]. This may also promote “regionalization of care”, a concept involving the establishment of systems where high-volume tertiary centers receive patients from surrounding regional hospitals based on well-defined criteria and transfer protocols [59]. This facilitates the prompt triage of PE patients and streamlines the delivery of care to high-risk patients. Previous data have demonstrated the inverse association between the annual cardiogenic shock volume (all phenotypes) and inpatient mortality, which have been linked to the more frequent use of standard supportive therapies including MCS in the higher volume centers [60]. Although the majority of hospitals in the United States offer acute cardiac care, there is a limited number of facilities equipped to provide cardiogenic shock care for PE-related shock, which entails advanced MCS, interventionalists, cardiothoracic surgeons, critical care experts, and specialized ancillary personnel. In addition, the expenses and complexity involved in maintaining MCS capabilities pose challenges for smaller centers with lower procedural volumes. Therefore, we emphasize the importance of promptly identifying and transporting patients with PE–cardiogenic shock to specialized Level I cardiac shock care centers, aiming to enhance survival rates among this challenging patient population. Those Level I dedicated cardiac shock care centers are high-volume, specialized centers equipped with cardiac catheterization, providing advanced MCS round the clock, seven days a week, along with on-site cardiothoracic surgery support [61].

Finally, since the ability to care for high-risk PE patients requires knowledge and training in cardiogenic shock and procedural competency regarding catheter-directed PE therapies, MCS cannulation and large bore access management, it is prudent that modern training for cardiovascular medicine and interventional cardiology should prepare the next generation of physicians for the timely hemodynamic assessment of these patients and prompt management, including the choice of reperfusion therapy, type and timing of mechanical circulatory support, and the management of procedural complications. In addition, interventional cardiology training programs should implement structured training for high-risk PE management, a multidisciplinary team approach, and the cannulation and management of MCS devices. This can be achieved through didactic sessions, simulation-based training, and hands-on experience under the guidance of experienced mentors. Training should also incorporate education on optimizing resource utilization, establishing effective systems of care, and engaging in the multidisciplinary team approach.

## 9. Conclusions

In summary, temporary MCS devices play a major role in the treatment of high-risk PE. The decision to implement MCS should be based on a thorough clinical evaluation tailored to each individual patient, with consideration given to the specific features of the device, as well as the center’s expertise. Commercially available devices reduce RA pressure and provide circulatory support ranging from 4 to 5 L/min. The timely initiation of MCS in patients exhibiting the early clinical signs of hemodynamic instability is paramount, as the insertion of MCS can be lengthy, especially in rapidly deteriorating patients. The dedicated PE response team implementing a stepwise approach could enhance the efficiency of the treating facility and potentially lead to better outcomes. Finally, large randomized controlled trials are needed to determine the effectiveness of MCS in high-risk PE.
